# Development of a topical tissue cross-linking solution using sodium hydroxymethylglycinate (SMG): viscosity effect

**DOI:** 10.1042/BSR20191941

**Published:** 2020-01-10

**Authors:** Jaya Mehta, Anna Takaoka, Mariya Zyablitskaya, Takayuki Nagasaki, David C. Paik

**Affiliations:** Department of Ophthalmology, Columbia University College of Physicians and Surgeons, New York, NY

**Keywords:** Cornea, formaldehyde releaser, Sclera, sodium hydroxymethylglycinate, Tissue cross-linking

## Abstract

Hyperviscosity agents are commonly used in ophthalmic formulations for improving corneal drug penetration by increasing tissue contact time. One such viscosity agent is hydroxypropyl methylcellulose (HPMC). HPMC has been used in riboflavin solutions for photochemical UVA cross-linking (CXL). Sodium hydroxymethylglycinate (SMG) is a small molecule formaldehyde releaser that can function as a therapeutic tissue cross-linker for corneal and scleral applications. The present study was undertaken in order to study formulation factors using HPMC and SMG that could positively influence the cross-linking effect in these ocular tissues. Formulations containing 10 mM SMG and 100 mM sodium bicarbonate were prepared with varying HPMC concentrations from 0 to 4.4%. Their cross-linking effects on porcine and rabbit eyes were measured using differential scanning calorimetry (DSC), expressed as the change/difference in melting temperature (Δ*T*_m_) compared with the control. SMG in 4.4% HPMC solution resulted in Δ*T*_m_ of 6.3 ± 1.21, while other concentration showed no differences in *T*_m_ shift on porcine cornea. In *ex vivo* rabbit cornea, there was a trend toward an increasing cross-linking effect with higher viscosity albeit mild differences. While a significant *T*_m_ shift was observed in porcine and rabbit sclera, there was no difference in effect of cross-linking between four HPMC concentrations. Increasing the HPMC concentration does not negatively affect the cross-linking efficacy attributed by SMG and could still be a positive cross-linking enhancer by virtue of increasing tissue contact time in a dynamic biological system. This information will be useful for planning further animal and human studies.

## Introduction

Keratoconus is being managed successfully with ultraviolet-A – riboflavin photochemical corneal cross-linking (known as ‘CXL’) throughout the world, which has revolutionized the field of corneal ectasia and related disorders. The treatment method utilizes riboflavin and UVA radiation [1]. The Dresden procedure, developed by Wollensak et al. in 2003, is used widely and requires removal of the epithelial layer, pre-soaking with riboflavin photosensitizer in 20% high molecular weight dextran (T500), and irradiation with UVA (355–365 nm max) light [2]. There are several reasons, however, to consider the development of topical cross-linking solutions to augment or replace the Dresden procedure, since the procedure does produce post-procedural corneal haze [3], keratocyte apoptosis [4] and changes in corneal microstructure [5,6]. This evidence, in addition to the pain and possible post-operative infection associated with de-epithelialization related to riboflavin’s inability to reliably penetrate the corneal epithelium, points toward the need for a less invasive treatment [7,8]. The procedure is also restricted to corneas with a thickness of 400 microns or more [8], requiring the disease to be detected and treated in its early stages, which in itself is an issue due to the difficulty in diagnosing keratoconus [9,10]. Hence, the realm of possibilities for providing an effective treatment that is efficient, but also safe for patients, remains open to new ideas and interventions.

Previously, our (Babar 2015) screening studies for potential cross-linking agents demonstrated that SMG induces significant cross-linking effects at a pH of 8.5 with an intact epithelium [11]. The small size of SMG (molecular weight = 127 Da) and hydrophilicity suggest that the molecule can penetrate the corneal epithelium via paracellular pathways and support its use as an ‘epi-on’ corneal cross-linking agent. Chemically, SMG is a glycine molecule with a hydroxymethyl component attached to the N terminal. The preservative ability of SMG is exploited for use in various products including shampoos, baby wipes, cleaning agents, among other household products [12]. The ubiquity of SMG in everyday products does reflect its safety at low concentrations.

For our studies, SMG was incorporated into a formulation containing hydroxy propyl methyl cellulose (HPMC), which is a polymer that has been used to form micro-emulsions to deliver drugs in a more stable manner [13]. HPMC is present in various commercially available eye drops and has been found to be stable, non-toxic and malleable with respect to its viscosity [14].

Ophthalmic preparations that are topically applied need to be formulated to have both maximal ocular contact time as well as corneal permeability [15]. A formulation will have maximum bioavailability when it is able to cover a larger surface area with the active agent; a means of doing this is by increasing its viscosity [16]. Viscosities up to 20 cP are well tolerated by the eye and can increase tissue bioavailability of the agent. Above a viscosity of 20 cP, there is a decrease in drug efficacy due to increased drainage, as resulting increased irritation will induce reflexive tearing [16]. Along with viscosity, pH is another key component of a topical formulation; pH can impact bioavailability via reflex blinking due to irritation [16]. Ideally, the pH should be within or close to the normal physiological range, as tears have buffer capacity that help reduce irritation and avoid total clearance of drug due to excess tear generation. However, this physiological range, which is matched to that of the lacrimal fluid, is not suitable for the activity of several drugs. Hence, a buffer that allows for a balance between maximal drug activity and minimal eye irritation should be chosen [16]. The active agent itself needs to be able to physically permeate through the epithelial and stromal layers. Therefore an amphipathic, small molecule is an ideal candidate [17]. These findings have formed the basis of our study. In order to facilitate pre-clinical live animal studies and in anticipation of human clinical trials, the present study focuses on creating an ideal formulation for SMG delivery into the eye for the expressed purpose of cross-linking tissues as a treatment for corneal ectasias and scleral conditions. In order to determine conditions to facilitate the most cross-linking effect in tissue, we manipulated viscosity within the formulation. The present study is a companion study to one that was recently published in Bioscience Reports in which factors affecting formaldehyde release from cosmetic preservatives (including SMG) were studied [18].

## Materials and methods

### Chemicals

Sodium hydroxymethylglycinate (Suttocide™ A, CAS #70161-44-3) solution was obtained from Ashland Inc. (Columbus, OH, U.S.A.). Hydroxypropyl methyl cellulose (HPMC, CAS #9004-65-3, 15cP at 2% in water @ 20°), protease inhibitor tablets (part# S8820) and sodium bicarbonate (NaHCO_3_, CAS# 144-55-8) were purchased from Sigma-Aldrich Corp. (St. Louis, MO, U.S.A.). Dulbecco’s phosphate-buffered Saline (DPBS) was purchased from VWR (Radnor, PA, U.S.A.). All chemical solutions and buffers were prepared fresh using Millipore water (double distilled, deionized water, *ρ* = 18.2 MΩcm at 25°C) on the day of the experiment.

### Tissue cross-linking

We performed chemical TXL with various concentrations of HPMC solutions (0, 1.1, 2.2 and 4.4%) and tested for differences in SMG cross-linking effectiveness on enucleated porcine globes purchased from Clements Food Group (Hatfield, PA). Eyes were kept frozen until the day of the experiment and thawed to room temperature prior to use. As a comparison, cross-linking experiments were performed using *ex vivo* rabbit eyes. Intact cadaveric rabbit heads with clear corneas were obtained from the local abattoir within an hour post-mortem and eyes were enucleated prior to the treatment. This work was exempted from IACUC monitoring, and the ethical approval was not required as only cadaveric tissues/samples were utilized. The formulation contained various concentrations of 15cP HPMC, while keeping the final SMG concentration at 10 mM and NaHCO_3_ at 100 mM. The concentration of 10 mM SMG was chosen based on the results from a previous rabbit cornea study in which this concentration was determined to be effective yet non-toxic [19]. Each formulation was prepared freshly on the day of the experiment. Each eye was placed in a 50 ml falcon tube, and 5 ml of the solution was added. Then, eyes were incubated for 2 h at room temperature. After the incubation period, the eyes were rinsed using DPBS. Three pieces of approximately 3 mm × 3 mm corneal tissue were obtained from a central strip of cornea, and multiple scleral 4 mm × 4 mm pieces were dissected out from each globe. Dissected corneal and scleral pieces were then subjected to differential scanning calorimetry (DSC, see below for the detailed procedure) analysis to determine their thermal denaturation temperature, a measurement of cross-linking efficacy.

### Differential scanning calorimetry (DSC)

Following the incubation, dissected corneal and scleral pieces were soaked in protease inhibitor solution. Prior to placement into pre-weighed 50 µl aluminum pan (Perkin-Elmer part# B0169321), dissected pieces were carefully blotted in a standardized, repetitive manner on a double-folded paper towel to remove excess solution. It should be noted that amounts under 2 mg have smaller signal to noise ratios and as such, can complicate thermogram peak analysis. Residual water in the pan can shift the thermogram downward since water has a high heat capacity. Also of importance is a flattening of the sample onto the bottom of the pan. This maximizes uniform heat transfer from the pan to the tissue and can affect the margin of error in readings. Then, pans were immediately hermetically sealed using a DSC pan sealing press (Perkin-Elmer part# B0139005), preventing tissue dehydration due to evaporative losses, and loaded into the DSC Autosampler.

Thermal denaturation temperature (*T*_m_) of all samples was measured using a Perkin-Elmer DSC 6000 Autosampler (part# N5370217, Waltham, Massachusetts, U.S.A.) with Pyris software (version 11.0). The samples were heated from 50 to 80°C at a scan speed of 1°C/min, which was determined to be an optimal scan speed based on preliminary runs. Denaturation curves representing differential heat flow over time were generated. DSC heat flow endotherm data was then analyzed by interpolation of a segment of the curve containing the major endotherm. The maximal transition temperature point was then identified using the Pyris data analysis peak search function. The instrument has an expected margin of error of ±0.3°C based on preliminary runs and is consistent with the manufacturer’s specifications. Less stable fibrils will result in a lower *T*_m_ and likewise, more stable fibrils induced by the cross-linking treatment will raise the *T*_m_. T_m_ has been used as a surrogate assay for tissue cross-linking efficacy, in lieu of mechanical testing, for many decades in the biomaterials world and we have applied to this simple and efficient technique to our therapeutic tissue cross-linking studies previously [19].

### Statistical analysis

For all porcine eye globe experiments, *T*_m_ of each treated tissue sample subtracted by the control *T*_m_ resulted in variable Δ*T*_m_, which is the mean of the control samples run that day. In order to compare the mean differences in Δ*T*_m_ between the different HPMC concentrations, linear mixed models were fitted to the outcome variable, where the predictors were the relevant concentration and the day of the experiment. This model allowed for variances to be determined between the relevant concentration groups by the inclusion of random intercepts for animals and controlled for correlation of observations that could be due to tissue from the same animal. Due to the nature of the *ex vivo* cadaveric system used for tissue cross-linking experiments, each cadaver provided the treated eye and contralateral control, and tissue samples were thus subjected to paired test. To that end, we have calculated Δ*T*_m_ by taking the average of the *T*_m_ values for the control (left) eye of a rabbit, then subtracted the average *T*_m_ of that rabbit’s control eye from the *T*_m_ values of the treated (right) eye. To compare the mean Δ*T*_m_ between HPMC concentration groups, we fit linear mixed models, where the outcome was the Δ*T*_m_ and the predictors were indicator variables for the HPMC concentration group. The model included random effects for rabbit. Then to test whether there were any mean differences between HPMC concentration groups, we performed an overall test, using a Wald test. Significance of all statistical tests were based on an alpha value of 0.05 (*P* ≤ .05). All Δ*T*_m_ values are reported in the form of mean value followed by standard error.

## Results

**Figure 1:**
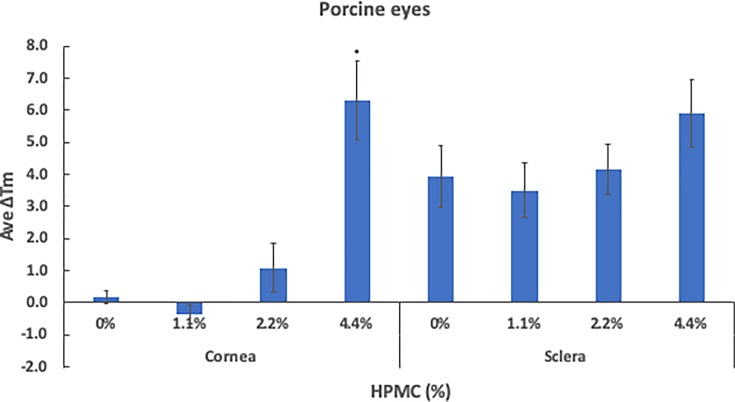
Two-hour treatment of whole porcine eye with 10 mM SMG in various 15cP HPMC concentrations Formulations containing different concentrations of 15cP HPMC (0, 1.1, 2.2, 4.4%) with 10 mM SMG were compared for their effect on cross-linking in cornea and sclera, after whole porcine globes were subjected to a 2-h incubation. Control tissue was prepared in an identical fashion but without SMG. Δ*T*_m_ represents the average shift in denaturation temperature to the control, after treatment. Cornea and sclera pieces were resected immediately after the incubation period. Asterisks (*) indicate significance (*P* < 0.05) based on non-paired test performed from three independent trials.

Various viscosity agents have been utilized in topical ophthalmic solutions, and 1.1% HPMC is a concentration often used in commercially available ophthalmic solutions. In the process of optimizing the drug formulation, our goal is to formulate a solution that has maximal ocular contact time as well as corneal permeability. Increasing the contact time of the drug in the solution can be achieved by increasing the viscosity of the solution; however, we were still uncertain whether increasing the concentration of HPMC could affect SMG efficacy. In our study, we have tested four different concentrations of HPMC solutions containing 10 mM SMG and 100 mM NaHCO_3_ (buffer) in order to determine the effect/interference of HPMC on tissue cross-linking. For preliminary experimentation, we have used the previously frozen porcine eyes. Cross-linking effect was measured using DSC. As shown in Figure 1, no significant difference in Δ*T*_m_ was observed in cornea that was treated with 0% (0.19 ± 0.20), 1.1% (−0.36 ± 0.29 versus 0% *P* = 0.07) and 2.2% (1.1 ± 0.75 versus 0% *P* = 0.245) HPMC solutions. Δ*T*_m_ of 6.3 ± 1.21 was observed using 4.4% HPMC solution (versus 0% *P* < 0.001), a rather dramatic difference by comparison to the other concentrations of HPMC tested. Sclera tissues were cross-linked more effectively by all of the preparations, resulting Δ*T*_m_ of 3.93 ± 0.97 with 0%, 3.51 ± 0.87 with 1.1% (versus 0% *P* = 0.742), 4.16 ± 0.78 with 2.2% (versus 0% *P* = 0.855) and 5.90 ± 1.05 with 4.4% HPMC solution (versus 0% *P* = 0.169). There were no statistically significant differences among the four HPMC preparations (Figure 1).

**Figure 2:**
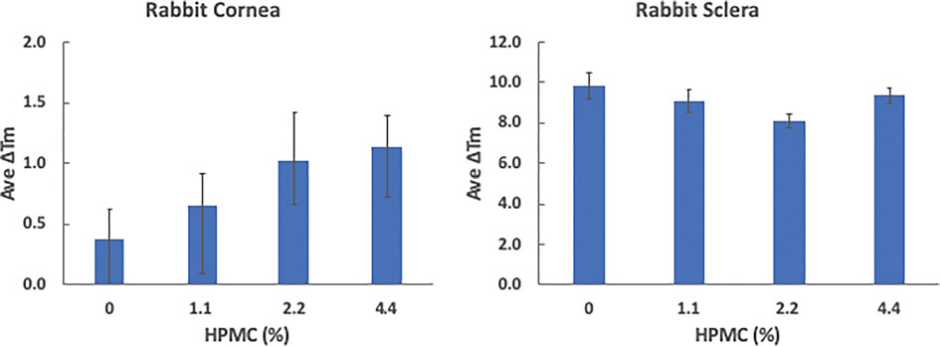
Two-hour treatment of whole rabbit eye with 10 mM SMG in various 15cP HPMC concentrations Formulations containing different concentrations of 15cP HPMC (0, 1.1, 2.2, 4.4%) with 10 mM SMG were compared for their effect on cross-linking in cornea and sclera, after enucleated rabbit eyes from *ex vivo* cadaveric rabbit head were subjected to a 2-h incubation. Control tissue was prepared in an identical fashion but without SMG. Δ*T*_m_ represents the average shift in denaturation after the incubation period. None of the groups reached statistical significance (*P* < 0.05) based on non-paired tests performed from three independent trials.

These results were further tested using *ex vivo* cadaveric rabbit eyes in which the epithelium layer was kept intact, and had the paired control eye for the treated eye from the same specimen. As was seen in the porcine experiment, a notable difference was that the sclera was again more effectively cross-linked, compared to cornea (Figure 2). There was no drastic increase in the cross-linking effect on cornea, with Δ*T*_m_ of 0.37 ± 0.26 with 0%, 0.65 ± 0.26 with 1.1% (versus 0% *P* = 0.503), 1.00 ± 0.42 with 2.2% (versus 0% *P* = 0.161) and 1.12 ± 0.27 with 4.4% HPMC solutions (versus 0% *P* = 0.115). Scleral cross-linking resulted in Δ*T*_m_ of 9.88 ± 0.66 with 0%, 9.07 ± 0.55 with 1.1% (versus 0% *P* = 0.459), 8.13 ± 0.34 with 2.2% (versus 0% *P* = 0.172) and 9.37 ± 0.40 with 4.4% HPMC solutions (versus 0% *P* = 0.634). Although there was no statistical significance in *ex vivo* samples, we have observed that higher viscosity solution stays longer in sub-Tenon’s space using ultrasound imaging; therefore, it might be more beneficial in the living animal study (data not shown).

## Discussion

Our study focuses on the development of a chemically and physiologically sound formulation using SMG, a cross-linking agent with a potential to become an alternative therapy for strengthening corneal and scleral tissues. SMG is an effective compound to induce cross-linking and its effects are directly proportional to its concentration within the formulation. However, maintaining a balance between toxicity and efficacy is key, in order to maximize the positive effects and minimize the adverse effects. This can be done by increasing the contact time between SMG and the tissues that require cross-linking. We have previously shown that 10 mM (1/4 concentration of EU max allowed concentration) SMG is a safe and effective compound that can induce cross-linking and remain non-toxic to endothelial cells on de-epithelialized corneal tissue during a 30-min exposure [19]. This is the reason that 10 mM SMG was chosen for the present work. By keeping the SMG concentration constant, our goal was to determine the effect of varying viscosity (by changing the concentration of HPMC) on the cross-linking effect of the formulations.

Viscosity is a critical component of ophthalmic preparations. There have been many viscosity enhancing agents used for drug delivery, but HPMC stands out quite clearly as being extremely efficacious and pliable [20–23]. There are various HPMC grades, and the grade and viscosity of HPMC are dependent on the differences in molecular weight (chain length) and the extent of hydroxypropyl and methyl substitutions [24]. We could possibly manipulate two properties of HPMC’s viscosity: concentration and grade. In a study of isoniazid (MW ∼137 Da) release from HPMC tablets [25], it was found that higher viscosity grades of HPMC decreased the rate of drug release from the tablet. They theorized this is due to the intra-polymer matrix interactions, particularly the disentanglement of polymer chains, which prevent effective drug release. These drug–polymer matrix interactions become greater with more branching present in higher grades of HPMC. Our expectation was that the higher HPMC grades, accompanied by greater degrees of branching, would cause more cross-liking effect in comparison with lower grade preparations, due to the ability of viscous agents to increase surface area of the drug in contact with the tissue. However, the effect was opposite – the lower grade HPMC, i.e. 15cP HPMC (which achieves a viscosity of 15cPs at 2% in water at 20°C, hence we call it ‘15cP HPMC’) showed higher cross-linking effect, in comparison with a higher grade HPMC (4000cP HPMC) (data not shown). These results are supported by other studies [26], indicating that the lower viscosity grades show better drug release and the release rate is indirectly proportional to the increases in polymer grade [25]. Chemically, perhaps the hyper-gel-like composition of the higher viscosities prevented the SMG from effectively penetrating the different layers within the cornea. The 2.2% 15cP viscosity differs chemically from the 1.1% 4000cP HPMC in the chain length, for instance. The 15 cP HPMC molecules have a lower chain length, whereas the 4000cP HPMC molecules have longer chain length. In theory, this lower dynamic viscosity of 2.2% 15cP HPMC would have a looser structure and could be making its matrix more porous to SMG, allowing for its release more effectively into the tissue. The higher dynamic viscosity, attributed to the 4000 cP HPMC, could in theory have a tighter molecular framework, preventing SMG from entering the matrix in the first place, as well as making it difficult for what does enter to exit. For these reasons, we have proceeded with our formulation study using a lower grade 15cP HPMC.

The high cross-linking effect observed with 4.4% in the porcine cornea might be attributed to various reasons. First, the condition of the epithelial layer may account for the differences in effectiveness in porcine and rabbit samples. The porcine eyes used in preliminary experiments were enucleated and frozen for days before being utilized for experiments. Therefore, the epithelium layer was not well preserved. Alternatively, the rabbit samples were used within an hour post-mortem, preserving the epithelium and clarity of the cornea. The rabbit cornea with an intact epithelium layer should have had a more regulated SMG diffusion to its corneal collagen layer; therefore, porcine cornea with disrupted epithelium was more susceptible to be cross-linked due to increased SMG diffusion. Second, the interspecies differences in the corneal collagen arrangements may be another reason for higher cross-linking effect. Meek and others have shown the variation in structural parameters, including interfibrillar spacing, in various species [27]. Formaldehyde, the active component released from SMG, reacts with various functional groups both intermolecularly and intramolecularly, yielding a variety of cross-linked products depending on the availability of the reactive sites [28–31]. Certain inter-species differences in the corneal collagen arrangements and/or spacings could possibly contribute to the differences in the degree of cross-linking.

A notable observation during our experiment was the differences in the responsiveness of cross-linking between the corneal and the scleral tissues. The cornea and sclera have similar collagen composition in mammals [32]; however, the anatomy of the collagen fibrils differ between the two with respect to collagen type V. Scleral tissue has a larger range of fibril diameter distributions containing type V collagen [32] and a higher amount of larger diameter fibrils [33,34]. The abundance and variability of larger diameter collagen fibrils, in conjunction with greater variability in inter-fibril spacing, results in greater cross-linking and increases the tensile strength of the tissue, allowing the sclera to play its biomechanical role in the eye. The cornea is well known for containing small uniform diameter fibrils with regular inter-fibril spacing, which cross-link to a lower degree [33], but therefore allows the cornea to remain elastic and transparent. This could potentially explain the differences seen between the two tissues in response to our formulation.

By comparison to the photochemical cross-linking technique, known as UV-riboflavin (CXL), it has been reported that human sclera was not as effectively cross-linked as human cornea with the energy density of 3 mW/cm^2^ and 370 nm illumination for 30 min [35,36]. Using the same technique of CXL, the mechanical rigidity was increased by 29% for sclera, while a drastic 329% increase was observed for cornea, in contrast to our results with SMG. Whereas, in porcine, the scleral cross-linking with CXL was more effective, compared with the cornea [35,36], similar to our results. Zhang et al. reported that the stiffness increased for rabbit sclera over rabbit cornea. This trend did not hold true for porcine and human sclera, however [37,38]. The reasons for these interspecies and inter-tissue inconsistencies are unclear, but these could be explained by variations in UV penetration of scleral tissue, scleral permeability of riboflavin solution and structural differences among different species [37,38].

HPMC has been effective in the delivery of nanocrystals to the eye [39]. Even though SMG is smaller in size than a typical nanocrystal (∼300 Da), it is possibly being released from the polymer in a similar manner. HPMC has been used to make ophthalmic inserts and films, which could be a possible method for our formulation to be applied [40,41]. Using an ointment is by far the most cost-effective solution, but an insert or a film would increase contact time, hence increase bioavailability and stability of the drug more efficiently. In our study, we limited the concentration of HPMC to 4.4% that is 4× the typical HPMC concentration of riboflavin-UVA photochemical cross-linking solution (i.e. 1.1%). Furthermore, it should be noted that as the solutions become increasingly more viscous, they also become increasingly more difficult to work with from a mixing and pipetting standpoint. It is for this reason that we limited the HPMC concentration to 4.4.%.

Our HPMC experiments using porcine and rabbit eyes indicate that increasing HPMC concentration would not hinder the effectiveness of SMG’s ability to cross-link tissue. This could be useful information in optimizing the formulation. It would be possible to use any viscosity levels (between 0% and 4.4%) to formulate the SMG solution. Considerations should be made to maintain the formulation’s cross-linking effect and also adjust the viscosity depending on the comfort level of the solution to the eye. The least viscous solution (0% HPMC) would have the shortest contact time, whereas a more viscous solution has longer contact time but may cause discomfort on patients’ eyes, causing frequent blinking after application on the cornea [16]. We are currently examining the effect of SMG cross-linking by injecting the SMG formulation in the posterior sub-Tenon’s space as a potential therapy or preventative measure for progressive myopia. For sub-Tenon’s space injections, there might be a benefit to use a high viscosity solution. We have observed that the higher the viscosity, the longer the formulations containing the drug stayed localized in the site of injection. The injected SMG formulation remains for up to 45 min to an hour, with a definite increase in residence time when compared with a saline injection (Unpublished). The present study provides valuable information to further optimize the formulation to effectively deliver the drug to the site of interest and induce cross-linking effects.

## Abbreviations

cP: centipoise
DSC: differential scanning calorimetry
FA: formaldehyde
FAR(s): formaldehyde releasing agent(s)
HPMC: hydroxypropylmethylcellulose
SMG: sodium hydroxymethylglycinate
TXL: tissue cross-linking
